# Surgical outcomes of one-stage resection for synchronous multiple primary lung adenocarcinomas with no less than three lesions

**DOI:** 10.1186/s13019-021-01647-z

**Published:** 2021-09-20

**Authors:** Rirong Qu, Dehao Tu, Wei Ping, Yixin Cai, Ni Zhang, Xiangning Fu

**Affiliations:** Department of Thoracic Surgery, Tongji Hospital, Tongji Medical College, Huazhong University of Science and Technology, Wuhan, 430030 People’s Republic of China

**Keywords:** Synchronous multiple primary lung adenocarcinomas (SMPLA), synchronous multiple primary lung cancers (SMPLC), Surgical outcome, One-stage

## Abstract

**Background:**

More and more synchronous multiple primary lung adenocarcinomas (SMPLA) have been diagnosed and surgical treatment has become the mainstay of treatment for them, but there are few reports on the surgical outcome of patients with ≥ 3 lesions who underwent surgical resection. Therefore, we summarized and analyzed the clinical characteristics and surgical outcomes of these patients, hoping to provide some experience in the diagnosis and treatment.

**Methods:**

Clinical characteristics and treatment outcomes of patients with ≥ 3 lesions who have been diagnosed as SMPLA and underwent surgical resection in our hospital from March 2015 to July 2019 were retrospectively reviewed.

**Results:**

Twenty-eight patients, 20 females and 8 males, with a mean age of 57.7 ± 5.69 (45–76) years, were finally included. A total of 95 lesions, 86.4% were ground-glass opacity (GGO) lesions (pure-GGO,45.3%; mixed-GGO,41.1%); 51 lesions had EGFR mutations and the mutation rate of invasive adenocarcinoma was significantly higher than that of other pathological subtypes (P < 0.001); the mutation rate of mGGO was also significantly higher than that of pGGO and solid nodule (SN) (P < 0.05). Four and 24 patients respectively underwent bilateral and unilateral surgical resection. The surgical procedure was mainly sublobar resection, and no severe postoperative complications or deaths occurred. After a median follow-up time of 32.2 months, the rates of overall survival and disease-free survival at 3 years were 94.7% and 88.9%, respectively.

**Conclusions:**

For SMPLA with ≥ 3 lesions, one-stage resection may be safe and feasible, and surgical procedure was mainly sublobar resection as far as possible, which can yield satisfactory prognosis. EGFR mutation testing should be used routinely in the diagnosis and treatment of patients with SMPLA, especially in the presence of mGGO and invasive adenocarcinoma.

## Introduction

With the popularization of lung cancer screening and the advancement of modern imaging technology, especially the widespread application of high-resolution computed tomography (HRCT) and positron emission tomography-computed tomography (PET-CT), more and more synchronous multiple primary lung cancers (SMPLC) are detected. The incidence of SMPLC ranges from 0.2 to 20% [1–3], of which 40.3 to 91.3% are SMPLA [4–7]. Although several studies [6, 8–12] have shown that surgical resection is the treatment of choice for these patients due to a good prognosis, there are still controversial issues related to the diagnosis, treatment, and prognosis of patients with SMPLC. In previous studies of SMPLA [8, 11, 13], patients with double primary lung adenocarcinoma accounted for more, but few studies have reported what surgical approach should be adopted and what is the effect of simultaneous surgical resection for SMPLA with ≥ 3 lesions. In the present study, we reviewed the clinical data and follow-up results of these patients who underwent single-stage surgery in our center to further determine the clinical characteristics of these lesions and to evaluate the efficacy of surgical treatments, hoping to provide our experience in the diagnosis and treatment of these patients.

## Patients and methods

### Patients

This study retrospectively analyzed the clinical data of patients with SMPLA who underwent simultaneous surgical resection in the thoracic surgery department of Wuhan Tongji Hospital between March 2015 and July 2019. The criteria for diagnosis of SMPLA in this study are based on the Martini-Melamed criteria [14] and incorporate elements of the new international multidisciplinary lung adenocarcinoma classification [15]: (1) major histologic subtypes of tumors are significantly different; (2) major histologic subtypes are similar, but all tumors have lepidic growth component to a certain proportion, or immunohistologic features or genetic profiles of tumors are different. The inclusion criteria were as follows: (1) number of tumors ≥ 3; (2) postoperative pathology of the patient's lesions were all lung adenocarcinoma; (3) the patient did not have adjuvant therapy before surgery; (4) cardiopulmonary function was acceptable and could tolerate surgery; (5) no previous history of tumors; (6) no distant metastases on preoperative examinations, which included chest CT scans, abdominal CT or ultrasonographic examinations, brain CT or magnetic resonance imaging, and whole body bone scans. The exclusion criteria were as follows: (1) incomplete patient data information; (2) the postoperative pathology of the lesion is not lung adenocarcinoma. This study was approved by the institutional review board of Tongji Medical College of Huazhong University of Science and Technology and consent was given by all patients before their clinical records were used.

### Surgical procedure

All procedures were performed with intravenous inhalation combined with anesthesia + double lumen endotracheal intubation. The surgery was performed using a 3 cm small single-port approach: a 3 cm incision was made between the 5th ribs in the mid-axillary line of the patient's surgery side to place a thoracoscope, an elbow laparoscopic suction device, electrocoagulation hooks, and a bipartite clamp was placed to hold the lung lobe if necessary. At the end of the operation, two pigtail catheters were placed to drain pleural effusion. In bilateral surgery, one side of the surgery is completed and the contralateral surgery is performed in the same way. In order to ensure the R0 resection of the tumor, we perform three-dimensional reconstruction through chest-enhanced CT before surgery to initially estimate the specific location of the lesion and the extent of the lesion to be removed; for small diameter and peripheral lesions, we will use preoperative percutaneous methylene blue staining location under the guidance of CT or guided by electromagnetic navigation. Specific surgical procedures and strategies for selecting the extent of surgical resection are described in our previous studies [16, 17].

### Follow-up

Follow-up was performed by outpatient or telephone. The follow-up time was calculated from the day after surgery and was followed up until October 2020. In the first year after surgery, chest CT, tumor markers and abdominal ultrasound were reviewed every 3 months; in the second year after surgery, the above indicators were reviewed every 6 months; the above indicators were reviewed annually after postoperative three years.

### Statistical methods

Measured data were expressed as mean ± standard deviation (SD) and differences between groups were analyzed by t-tests. Counted data were expressed as number or percent, and differences were analyzed using X^2^ or Fisher’s exact tests. The above data was analyzed using Statistical Product and Service Solutions (version 23; SPSS Inc., Chicago, IL, USA). OS and RFS were performed using GraphPad Prism software version 7.0. P < 0.05 was considered statistically significant.

## Results

### Clinical data of patients and tumors

Clinical data of patients and tumors are shown in Tables 1 and 2 respectively. Twenty-eight patients, 20 females and 8 males, with a mean age of 57.7 ± 5.69 years (range 45–76 years) were finally included. Eleven patients had underlying diseases, and only 5 patients had a family history of tumors; except for 3 patients who had elevated CEA levels, CEA levels of the remaining patients were normal. All patients had acceptable preoperative cardiopulmonary function. The number of lesions was 3 in 20 patients (71.4%), 4 in 5 patients (17.9%), and 5 in 3 patients (10.7%). Twenty-five patients had GGO lesions, and 3 patients had only solid nodules. The lesions of 22 patients were located in different lobes, while the lesions of only 6 patients were located in the same lobe. A total of 95 lesions with an average diameter of 15.89 ± 8.97 mm; 86.4% were GGO lesions (pGGO, 45.3%; mGGO, 41.1%). Among 28 patients, the highest pathological T stage was mainly pT1 (92.9%) and only one patient had N2 lymph node metastasis. Twenty-two patients had EGFR mutations, and only one patients received adjuvant therapy due to N2 lymph node metastasis.

**Table 1:** Clinical data of patients

Variables	Number (%)	Mean value
Age (years)		57.7 ± 5.69
≥ 60	11 (39.3)	
< 60	17 (60.7)	
Sex		
Male	8 (28.6)	
Female	20 (71.4)	
Smoking status		
Current and former	8 (28.6)	
Never	20 (71.4)	
Family history of tumor		
Yes	5 (17.9)	
No	23 (82.1)	
Comorbidity		
Yes	11 (39.3)	
No	17 (60.7)	
Preoperative CEA level		3.38 ± 1.38
≥ 5.0 ng/ml	3 (10.7)	
< 5.0 ng/ml	25 (89.3)	
Ejection fraction		61.16 ± 4.02
≥ 60	16 (57.1)	
55–59	12 (42.9)	
FEV1(L)		2.68 ± 0.42
p-FEV1%		96.95 ± 24.16
≥ 100	8 (28.6)	
80–100	10 (35.7)	
≤ 80	10 (35.7)	
Distribution of tumors		
Unilateral	24 (85.7)	
Bilateral	4 (14.3)	
Number of tumors		
3	20 (71.4)	
4	5 (17.9)	
5	3 (10.7)	
Highest pT		
T1	26 (92.9)	
T2	2 (7.1)	
Highest pN		
N0	27 (96.4)	
N1-2	1 (3.6)	
Adjuvant chemotherapy		
Yes	1 (3.6)	
No	27 (96.4)

**Table 2:** Clinical data of tumors

Variables	Number(%)	Mean value
Total number of tumors	95	
Tumor characteristics (mm)		
pGGO	43 (45.3)	9.60 ± 4.23
mGGO	39 (41.1)	16.37 ± 6.35
SN	13 (13.6)	24.01 ± 11.67
Tumor type pattern per patient		
Multiple pGGO	5 (17.9)	
Multiple mGGO	3 (10.7)	
pGGO + mGGO	13 (46.4)	
SN + GGO	4 (14.3)	
Multiple SN	3 (13.7)	
Location of tumors		
RUL	44 (46.3)	
RML	14 (14.7)	
RLL	12 (12.6)	
LUL	17 (17.9)	
LLL	8 (8.5)	
Location of lobe		
Same lobe	6 (21.4)	
Different lobe	2 (7.1)	
Combined lobe^a^	20 (71.5)	
Size of tumors (mm)		15.89 ± 8.97
≤ 10 mm	52 (54.7)	
< 10 mm, ≤ 20 mm	32 (33.7)	
> 20 mm	11 (11.6)	
Histology in all tumors		
AIS	27 (28.4)	
MIA	17 (17.9)	
IA (WD/MD/PD)	51 (9/36/6)(53.7)	
EGFR in tumors per patient		
WT	6 (21.4)	
Mutation	22 (72.6)	

SN, solid nodule; pGGO, pure ground-glass opacity; mGGO, mixed ground-glass opacity;

RUL, right upper lobe; RML, right middle lobe; RLL, right lower lobe; LUL, left upper lobe; LLL, Left lower lobe; ^a^More than 2 cancers, at least 2 tumors were located at the same lobe and the other or others located at the different; AAH, atypical adenocarcinoma hyperplasia; AIS, adenocarcinoma in situ; MIA, minimally invasive adenocarcinoma; IA, invasive adenocarcinoma; WD, well-differentiated; MD, moderately-differentiated; PD, poorly differentiated

### Surgical data of patients

Four and 24 patients respectively underwent bilateral and unilateral surgical resection. The surgical procedure was mainly sublobar resection, and no severe postoperative complications or deaths occurred. The average operation time was 196.74 ± 71.39 min, the average intraoperative blood loss was 260.65 ± 208.25 ml, the mean postoperative daily drainage of chest tube was 159.34 ± 28.74 ml, the mean postoperative chest tube duration was 6.12 ± 3.21 days, and the average postoperative hospital stay 10.51 ± 4.94 days. Details of surgical procedure are described in Table 3.

**Table 3:** Surgical data of patients

Variables	Number
*Surgical procedure*	
Unilateral	24
Wedge resection-wedge resection	4
Segmentectomy-wedge resection	5
Segmentectomy-segmentectomy	1
Lobectomy-wedge resection	4
Lobectomy-segmentectomy	2
Lobectomy-lobectomy	2
Single lobectomy	6
Bilateral	4
Wedge resection-wedge resection	1
Wedge + wedge resection-segmentectomy + wedge resection	1
Segmentectomy-wedge resection	1
Lobectomy + segmentectomy-wedge resection	1
*Perioperative results*	
Operation time (min)	196.74 ± 71.39
Intraoperative blood loss (ml)	260.65 ± 208.25
Postoperative chest tube duration (day)	6.12 ± 3.21
Daily drainage of chest tube (ml)	159.34 ± 28.74
Postoperative hospital stay (day)	10.51 ± 4.94

### EGFR mutation in 95 tumors of 28 patients with MPLA

Postoperative EGFR detection of all lesions revealed that 51 lesions had mutations, mainly L858R and 19DEL, and their mutation rates were 30.5% and 16.8%, respectively. Among the different pathological subtypes, the mutation rate of invasive adenocarcinoma was significantly higher than that of other pathological subtypes (P = 0.000); the mutation rate of mGGO was also significantly higher than that of pGGO and solid nodule (SN) (P = 0.039); regardless of whether it is in different pathological subtypes or different lesion types, there is no significant difference in the mutation rate of L858R and 19DEL (P > 0.05). The results of EGFR mutation are presented in Table 4.

**Table 4:** EGFR mutation in 95 tumors of 28 patients with MPLA

Variables	Total(n)	AIS	MIA	IA	*P* value	pGGO	mGGO	SN	*P* value
Wild	44	19	14	11		25	12	7	
Mutation	51	8	3	40	0.000	18	27	6	0.039
L858R	29	7	1	21		7	18	4	
19DEL	16	1	0	15		8	6	2	
L858R/Other^a^	2	0	0	2		0	2	0	
19DEL/Other^a^	1	0	1	0		1	0	0	
Other^a^	3	0	1	2	0.057	2	1	0	0.372

AIS, adenocarcinoma in situ; MIA, minimally invasive adenocarcinoma; IA, invasive adenocarcinoma; SN, solid nodule; pGGO, pure ground-glass opacity; mGGO, mixed ground-glass opacity; ^a^Refers to other rare mutations including L861Q, G719X, 20Ins and T790M

### Disease free survival and overall survival of all patients

**Fig. 1:**
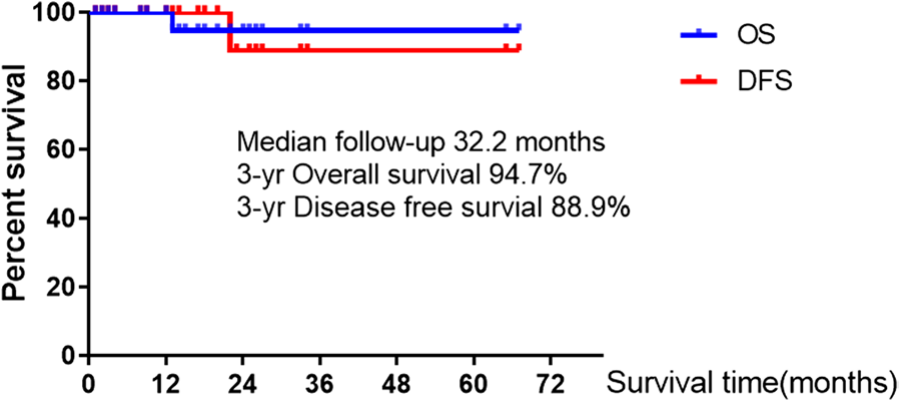
Disease free survival and overall survival of all patients

We followed up all patients after surgery and no follow-up was lost. As of October 10, 2020, the median follow-up time is 32.2 months. Except for two patients who developed distant metastases, one of whom died of extensive pleural metastases, all patients did not develop new lesions or metastases and are currently alive, and the 3-year overall and disease-free survival rates were 94.7% and 88.9%, respectively (Fig. 1).

## Discussion

Since it was first reported by Beyreuther in 1924 [18], SMPLC are not rare diseases, and its incidence continues to rise due to advances in imaging technology and an increasing aging population. Compared with intrapulmonary metastasis, the treatment options and prognosis of SMPLC are completely different. Therefore, the diagnosis, treatment and prognosis evaluation of SMPLC are particularly important. However, the widely used diagnostic criterion is still Martini-Melamed criterion [14], which is based primarily on the histological characteristics of the tumor. With the increase in related study on SMPLC, in 2003, 2007 and 2013, the American College of Chest Physicians (ACCP) successively revised and updated these criteria to include the use of molecular genetics of tumors [19]. Takuwa and colleagues [20] reported that a patinet with SMPLA involving multiple lobes of the same lung had mediastinal lymph node metastasis, and EGFR mutation of tumors are completely different. There was also a similar case with mediastinal lymph node metastasis in this study in which all tumor pathology types were lung adenocarcinoma, but the EGFR mutations were completely different. Such patients should be considered as MPLC, because adenocarcinomas often comprised various histologic subtypes (acinar, papillary, etc.), which suggests that these tumors have different origins [21]. Therefore, the diagnosis of SMPLA should take into account other factors of the tumor, such as major histologic subtypes, lepidic growth component to a certain proportion, immunohistologic features and genetic profiles of tumors.

Surgery is still the first treatment for multiple primary lung cancers, but the specific surgical method is still controversial. The key to the dispute is whether sublobar resection can achieve a good prognosis. Several studies [8, 11, 13, 22] have shown that for SMPLA, lobectomy should be used as much as possible for the primary lesions, while sublobar resection (segmentectomy or wedge resection) for the secondary lesions can be flexibly adopted, especially in patients with bilateral lung lesions so as to ensure adequate distance between tumor incision margins and to maximize the preservation of more lung function. Nakata et al. reported [8] that 26 patients with SMPLA, only 5 patients underwent lobectomy alone, and the 3-year OS and DFS were 92.9% and 77.9%, respectively. Ishikawa et al. also reported [11] that 93 patients with SMPLA, sublobar resection was used during surgery in 58% of patients, and the 3-year OS and RFS were 93.6% and 87% respectively. In the current series, since the number of lesions was ≥ 3 and most of them were distributed in different lobes, we tried to adopt a combined sublobar resection approach during surgery, and the OS and DFS at 3 years reached 94.7% and 88.9%, respectively, which was comparable to the results of the above study.

There is still no consensus on the choice of simultaneous or staged surgery for SMPLC, especially for bilateral SMPLC. Iino and colleagues reported [23] that patients who underwent bilateral surgical resection had better outcomes than those receiving combined surgical and chemotherapy or radiotherapy, but they advocate staged surgery for patients with bilateral lobectomy. Although staged surgery is more acceptable, one-stage surgery does offer several advantages. First, it addresses all lesions at once, reducing the patient's overall hospital and surgical costs; Second, it shortens the overall duration of anesthesia and surgery, shortening the length of hospital stay; Third, and most importantly, it prevents tumor progression and reduces patient anxiety. Previous studies [11, 13, 23–27] have shown that single-stage surgery is safe and feasible for bilateral SMPLC, but there is a requirement for patient selection. In this study, all patients recovered successfully after surgery and four patients underwent simultaneous bilateral thoracoscopic surgery. Hence, simultaneous surgery is safe and effective, and it should be the treatment of choice, especially for those patients who are in good health and can tolerate simultaneous surgery.

For multiple primary lung adenocarcinoma, we should routinely test for EGFR mutations in all lesions. First of all, in the situation that the diagnostic criteria of SMPLC are still not perfect, EGFR mutation can be a good supplement to histological, imaging and morphological evidence of tumor, so as to better distinguish multiple primary lesions from metastatic lesions and provide patients with a more accurate staging; Secondly, EGFR mutation can provide evidence of targeted therapy for patients; Finally, compared with patients with squamous cell carcinoma, patients with lung adenocarcinoma are more likely to be detected with EGFR mutations. In the present study, we found that 22 patients (72.6%) had at least one lesion with EGFR mutations and 53.7% of 95 lesions had EGFR mutations, which may be related to the fact that patients in the present study were still predominantly non-smoking women (71.4%) and had predominantly invasive adenocarcinoma (53.7%). In addition, our results indicated that among the different pathological subtypes, the mutation rate of invasive adenocarcinoma was significantly higher than that of other pathological subtypes (P = 0.000); the mutation rate of mGGO was also significantly higher than that of pGGO and solid nodule (SN) (P = 0.039); regardless of whether it is in different pathological subtypes or different lesion types, there is no difference in the mutation rate of L858R and 19DEL. This is similar to the findings of M. Liu et al. [28].

The present study has some shortcomings. First, the nature of this study was a single-center retrospective study, and selection bias is unavoidable; Second, although the incidence of SMPLA with ≥ 3 lesions was not high, the sample size of this study was still small; Third, this study did not compare the prognosis of patients who underwent simultaneous surgical resection with those who did not undergo simultaneous surgical resection and those who did not undergo surgical treatment; Fourth, the postoperative follow-up of the patients was not long enough, and the 5-year survival still needs to be verified by the follow-up results. A larger sample size, multi-center study is needed to verify and supplement the current study in the future.

## Conclusions

In summary, EGFR mutation testing should be applied to the diagnosis and treatment of patients with SMPLA, especially for patients with mGGO or invasive adenocarcinoma. One-stage surgery may be safe and feasible and it can be considered as the treatment of choice for SMPLA with ≥ 3 lesions due to a promising prognosis.

## Data Availability

All data generated or analysed during this study are included in this published article.

## Abbreviations

SMPLA: Synchronous multiple primary lung adenocarcinomas
SMPLC: Synchronous multiple primary lung cancers
SN: Solid nodule
pGGO: Pure ground-glass opacity
mGGO: Mixed ground-glass opacity
RUL: Right upper lobe
RML: Right middle lobe
RLL: Right lower lobe
LUL: Left upper lobe
LLL: Left lower lobe
AIS: Adenocarcinoma in situ
MIA: Minimally invasive adenocarcinoma
IA: Invasive adenocarcinoma
WD: Well-differentiated
MD: Moderately-differentiated
PD: Poorly differentiated
